# Earl Spencer on the Gestation of Cows

**Published:** 1841-01

**Authors:** Earl Spencer


					ON THE GESTATION OF COWS.
BY THE RIGHT HON. EARL SPENCER.
[The following observations are highly valuable, and deserving the attention
of the physiologist and statistician. Their important analogical bearing on the
exact period of human gestation is obvious ; as they supply an element, on a
large scale, which can rarely be obtained in the other case, viz., the exact
period of impregnation.]
For the purpose partly of curiosity and partly because I thought the notions
entertained respecting the ordinary period of gestation of cows incorrect, I several
years since began to take notes, whenever a cow calved, of the length of time
she had been pregnant; and, having now the periods of gestation of 764 cows
taken in this way, I think a sufficient number of cases has been collected to
enable me to draw general conclusions from the observations which I have
made.
I shall begin by inserting a Table which will show how many cows producing
live calves have gone each of the different periods therein mentioned. The first
column shows the number of days of gestation; the second the number of
cows which have gone each period; the third and fourth columns show whether
VOL. XI. NO. XXI. 18
274 Medical Intelligence. [Jan.
the produce was a cow-calf or a bull-calf; the fifth, if it was twin cow-calves ;
the sixth, if it was twin bull-calves; and the seventh, if it was twins of different
sexes. For instance, if 279 is taken, it will appear that 32 cows went 279 days;
that 16 of them produced cow-calves, 11 of them produced bull-calves, 3 of them
produced twin cow-calves, none of them produced twin bull-calves, and 2 of them
produced twins of different sexes.
o $
220
226
233
234
235
239
242
245
246
248
250
252
253
254
255
257
258
259
262
263
266
268
269
270
271
272
273
274
275
?S -g
^ ?
is m
H
2
276
277
278
279
280
281
284
285
287
291
292
293
294
295
296
297
299
304
305
306
307
313
1
fi
?
O rt
O O
c 3
Eh w
From the inspection of this table it will be seen that the shortest period of
gestation, when a live calf was produced, was 220 days, and the longest 313 days;
but I have not been able to rear any calf produced at an earlier period than 242
days. Any calf produced at an earlier period than 260 days must be considered
decidedly premature; and any period of gestation exceeding 300 days must also
be considered irregular; but in this latter case the health of the produce is not
affected. It will also be seen that 314 cows calved before the 284th day, and
310 calved after the 285th; so that the probable period of gestation ought to be
considered 284 or 285 days, and not 270 as stated in the book upon Cattle, pub-
lished under the superintendence of the Society for the Diffusion of Useful
Knowledge.*
* In another work, however, entitled " British Husbandry," published under the
superintendence of the Society for the Diffusion of Useful Knowledge, the experiments
of M. Teissier, of Paris, on the gestation of cows, are recorded to have given the
following results:
21 calved between the 240th and 270th day, the mean term being 259g
544 ditto 270th ? 299th ? ? 282
10 ditto 299th ? 321st ? ? 303
" In most cases, therefore, between 9 and 10 months may be assumed as the usual
period; though with a bull-calf, she has been generally observed to go about 41 weeks,
and a few days less with a female." (Vol. ii., p. 438.)?F. Burke.
1841.] Books Received for Review. 115
It appears also that the number of breeding females is less considerably than
the number of males ; and to the number of males ought generally to be added,
as animals that will not breed, the females who are twins with males. I have
heard and believe that in some cases a cow-calf, twin with a bull, will breed ;
but in no instance in which I have bred twins of different sexes has the female
been a breeding heifer. The number of breeding heifers from these 764 cows
was 354 ; the number of bull-calves 422; and the number of heifers twin with
bulls, usually called fremartins, 11.
There is a prevalent belief among farming men, and I believe farmers, that,
when the time of gestation of a cow is longer than usual, the produce is gene-
rally a male calf. I must confess that I did not believe this to be the case, but
this table shows that there is some foundation for the opinion. In order fairly
to try this, the cows which calved before the 260th day, and those which calved
after the 300th, ought to be omitted as being anomalous cases, as well as the
cases in which twins were produced: and it will then appear that, from the cows
whose period of gestation did not exceed 286 days, the number of cow-calves
produced was 233, and the number of bull-calves 234; while, from those whose
period exceeded 286 days, the number of cow-calves was only 90, while the
number of bull-calves was 152.
Journal of the English Agricultural Society. Part ii., 1839.

				

